# The predictors of adaptive and maladaptive coping behavior during the COVID-19 pandemic: The Protection Motivation Theory and the Big Five personality traits

**DOI:** 10.1371/journal.pone.0258606

**Published:** 2021-10-19

**Authors:** Irena Pilch, Paulina Wardawy, Eryka Probierz

**Affiliations:** Institute of Psychology, Faculty of Social Sciences, University of Silesia, Katowice, Poland; College of Medicine and Sagore Dutta Hospital, INDIA

## Abstract

The aim of this study was to evaluate the predictive value of the constructs proposed by two influential theoretical frameworks: the Protection Motivation Theory (PMT) and the Big Five (B5) model of personality in predicting health-related coping behavior during the SARS-CoV-2 pandemic. Both adaptive (i.e., preventive) and maladaptive (i.e., avoidant behavior and wishful thinking) coping behavior was measured. The study was conducted during the first wave of the pandemic. The sample consisted of 397 persons from the general Polish population. The findings provided strong support for the PMT constructs’ predictive value, especially with regard to pandemic-related adaptive behavior. The B5 personality traits accounted for a small proportion of the variability of coping behavior, especially when maladaptive behavior was the outcome. The PMT model showed incremental validity over and above demographic variables and personality traits in predicting preventive behavior, avoidant behavior, and wishful thinking; however, the patterns of relationships differ across the types of coping behavior. According to the current results, to increase adherence to preventive measures during an epidemic, all the PMT constructs should be considered while persuasive communication to the public is formulated.

## Introduction

In March 2020, the World Health Organization declared a state pandemic caused by the SARS-CoV-2 virus leading to the COVID-19 disease [1]. Due to the lack of pharmacological agents to effectively treat COVID-19, and with the aim of avoiding health care system overload, many countries imposed restrictions to contain the number of infections [1, 2]. However, the effectiveness of the regulations introduced depended on the degree of compliance with them. Individuals differed considerably in their reactions to the restrictions: some treated the situation responsibly and observed the rules, while others did not respect the preventive measures, engaging in maladaptive behaviors [3–6].

Seeking the determinants of adaptive and maladaptive behaviors during the pandemic may contribute to a better understanding of how individuals function in a situation of serious threat to health and life [7]. Moreover, these results may make it possible to adopt measures to reduce the occurrence of maladaptive behaviors, as well as increase the likelihood of protective behaviors. Previous studies have shown that factors that may be significantly associated with protective behaviors undertaken during the pandemic include, among others, sociodemographic, personality, cultural, social variables, and variables related to perception of the situation [7–10]. The current study aims at investigating the relationships between (adaptive and maladaptive) coping behavior during the pandemic and a set of predictors derived from the Protection Motivation Theory (PMT) and the Big Five (B5) personality model.

### Sociodemographic and personality correlates of compliance with pandemic recommendations

Research has shown that sociodemographic variables were associated with the manner of perceiving the pandemic and with acceptance of the restrictions. Older individuals complied with the recommendations to a greater extent [5, 11] and perceived themselves as more susceptible to diseases [7, 8]. Women were more concerned about the COVID-19 pandemic [12], experienced more emotional problems [13], and were characterized by a higher perceived overall disease susceptibility [7] compared to men. Lower education and not working remotely were also associated with higher perceived disease susceptibility [7]. Additionally, living in rural areas, lower education and lower income were negatively associated with adherence to the recommendations, acceptance of preventive measures, and knowledge about COVID-19 [8].

Most studies analyzing the associations between health-related behaviors during the pandemic and personality traits have used the Big Five/Five-Factor Model of personality [14]. A connection has been demonstrated between agreeableness and adaptive behaviors–compliance with recommendations and restrictions [5, 6, 15, 16], support for the recommendations, and a positive opinion on the government’s measures adopted to combat the pandemic [7]. Openness to experience and conscientiousness were associated with resistance to stress resulting from the COVID-19 pandemic [17]. Conscientiousness was also associated with compliance with the recommendations [5, 15, 16, 18]. The research findings concerning neuroticism are inconclusive. Neuroticism has been shown to be associated with adherence to social distancing rules [16, 18] and hygiene rules [16]. Other studies in turn have shown that neuroticism was negatively linked to engaging in protective behaviors [15] and adherence to official guidelines [19].

Other studies sought links between behavior during the pandemic and lower-level personality traits. Individuals characterized by a sense of duty and those perceiving pandemic-related events negatively were more likely to comply with the restrictions [6]. Optimistic individuals and those trusting of other people experienced lower anxiety and engaged more often in preventive behaviors [9]. Research also suggests a link between the Dark Triad, consisting of narcissism, Machiavellianism, and psychopathy, and maladaptive behaviors involving non-compliance with the recommendations [4, 6, 16]. Fear of COVID-19 was also a predictor of compliance with the recommendations in force [20–22].

### Protection Motivation Theory

Adherence to pandemic-related recommendations can be considered at the individual level as displaying protective behaviors undertaken to preserve one’s health. Health behaviors are studied in different contexts–in relation to disease prevention or to early detection. Competing psychological theories offer different sets of variables to be considered in research and practice. Key theories include the following: health belief model, protection motivation theory, theory of planned behavior, social cognitive theory, health locus of control, and stage models of health behavior [23–26].

This study is based on the Protection Motivation Theory (PMT, [27]). According to this concept, protection motivation coordinates behaviors undertaken when confronting a threat–it stimulates, sustains, and guides actions aimed at protecting oneself from danger [24, 28–31]. Significant factors in the manner of responding to a threat include threat and coping appraisal. The PMT distinguishes adaptive and maladaptive responses to health risks. These responses result from the manner in which the threat is appraised and the perceived ability to cope with it. The two appraisals determine whether the individual engages in an adaptive or maladaptive action. Threat appraisal is based on perceived vulnerability of a person and severity of the threat. Coping appraisal consists of analyzing behaviors that may reduce the health threat–the analysis concerns perceived response-efficacy, perceived self-efficacy, and perceived response costs. Adaptive behaviors occur when the individual assesses as high the level of the threat, their vulnerability to the threat, the effectiveness of the behaviors, and their personal ability to engage in them, and when they assess as low the costs of engaging in such behaviors.

The PMT model has been used as a theoretical basis for research in previous epidemics, such as H1N1, H5N1, SARS [11, 32–34], as well as during the current COVID-19 pandemic [35–38]. PMT-based research provides a better understanding of human behavior in response to health threats such as the presence of infectious diseases. Factors that were particularly significantly associated with adherence to pandemic-related recommendations included the perceived effectiveness of the existing recommendations and perceived self-efficacy in following them [28, 32–34, 36, 37, 39]. Other factors linked with the motivation to adopt preventive behaviors included perceived level of disease severity and susceptibility to infection [32, 35, 38, 40, 41]. In conclusion, research taking into account the factors included in the PMT may support the development of interventions shaping desirable behaviors during the pandemic.

### Current study

The objective of the current study was to investigate to what extent the two groups of individual factors (cognitive-emotional evaluations and personality traits) can contribute to the prediction of health-related coping behavior during the COVID-19 pandemic. Two meaningful theories, i.e., the Protection Motivation Theory (PMT [27]), and the Five-Factor/Big Five (FFM/B5 [14]) model of personality, formed the basis of this research. The PMT was used as a social cognition model to predict health-related behavior. As was reported in the Introduction, the PMT includes a range of variables grouped in the threat appraisal and coping appraisal categories. These variables were found in previous studies to be important predictors of health-protective motivation and behavior [28, 29]. In turn, the FFM/B5 is one of the most influential theories of personality, and the motivational aspect of the FFM/B5 traits is well-established [42]. The FFM/B5 traits can predict a wide range of behaviors [43, 44], including health and coping behavior [45–48].

Because the PMT constructs can predict both motivation (i.e., intentions) and behavior, and our study was conducted in the middle of the first wave of the pandemic (when information about pandemic recommendations was commonly available and pandemic restrictions had already been introduced), we decided to measure actual behaviors rather than the motivation of future behavior. As in other PMT studies [24, 40, 49–52], both adaptive (i.e., rational problem solving) and maladaptive (i.e., avoidant behavior and wishful thinking) behaviors were measured. As in most applications of the PMT, we analyzed the additive effects of the PMT constructs on health behavior [24, 27–29]. So far, personality traits have been rather seldom investigated in the context of the PMT, despite their importance being valued in the PMT framework [27]. Including both cognitive-emotional and personality factors in this study enabled comparison of the predictive power of these two important groups of predictors of pandemic-related behavior. This also made it possible to establish incremental validity of the PMT beyond personality traits and sociodemographic factors.

The following research questions were addressed: (1) What are the relationships between the PMT constructs and adaptive and maladaptive coping behavior during the first wave of COVID-19 pandemic? (2) What are the relationships between the B5 personality traits and adaptive and maladaptive coping behavior during the first wave of the COVID-19 pandemic? (3) Do the PMT constructs have incremental validity over and above the B5 personality traits and sociodemographic factors in predicting pandemic-related coping behavior?

Taking into account theory and past research, the following hypotheses were formulated:

I. Hypotheses regarding relationships between the PMT constructs and coping behavior in the face of a pandemic threat:

H1a. Perceived vulnerability to and severity of COVID-19, fear of COVID-19, self-efficacy, and response-efficacy will be positive predictors of adaptive coping behavior.
H1b. Perceived response costs will be a negative predictor of adaptive coping behavior.
H2a Perceived vulnerability to and severity of COVID-19, self-efficacy, and response-efficacy will be negative predictors of maladaptive coping behavior (i.e., avoidant behavior and wishful thinking).
H2b. Perceived costs and fear of COVID-19 will be positive predictors of maladaptive coping behavior (i.e., avoidant behavior and wishful thinking).

The above hypotheses were formulated on the basis of the PMT [24, 27]. Putting them forward is also justified by a significant number of empirical studies, including both ones referred to the PMT [11, 28, 29, 32–36, 38–41] and ones based on other theoretical foundations [20, 21, 37].

II. Hypotheses regarding relationships between personality traits and coping behavior in the face of a pandemic threat:

H3a. Agreeableness and conscientiousness will be positive predictors of adaptive coping behavior.
H3b. Neuroticism will be a positive predictor of maladaptive coping behavior (i.e., avoidant behavior and wishful thinking).

The above hypotheses were formulated on the basis of the FFM/B5 personality model [14]. Putting them forward is also justified by the results of research on coping behavior [53], including several studies on epidemic behavior [5, 6, 15, 16, 18, 19, 54].

## Materials and methods

### Ethics statement

This study was reviewed and approved by Ethics Committee at the University of Silesia in Katowice prior to data collection. The participants provided their written informed consent to participate. The study was conducted in accordance with the Declaration of Helsinki.

### Participants and procedure

The study was conducted on 397 persons from the general Polish population, aged 18–72 years (*M* = 31.2, *SD* = 11.5), 46% of whom were men (*n* = 181). Thirty-nine percent of participants completed secondary education, 5% had vocational training, 16% had a Bachelor’s degree, and 40% had a Master’s degree. Most of the participants (60%, *n* = 201) were employed on a permanent basis, 27% were university students. Forty percent of participants declared themselves as single, whereas 60% were in formal or informal relationships. Twenty percent of the participants (*n* = 78) perceived a high risk of complications caused by COVID-19, and 39% (*n* = 154) perceived COVID-19 as a threat to their relatives.

The study was conducted from 16^th^ May to 15^th^ June 2020. Convenience sampling was used for data collection. The participants took part in an online survey. They were recruited using online advertisements on websites, forums, and social media platforms. The inclusion criteria were age (≥18) and consent to participate. The participants were informed about the aim of the study, and that participation was voluntary, anonymous, and without compensation. After that, they gave their written informed consent (for participation and using their answers for scientific purposes). They also asserted that they were of age. Then, they were asked to provide sociodemographic data and complete the questionnaires related to the PMT constructs (the PMQ and FCV-19S). The PMQ items were presented in random order. After that, the participants completed the preventive behavior scale (PBS) and the questionnaire assessing personality traits. Lastly, a set of items unrelated to the current study was provided. This part of the survey was intended to collect additional data about the daily schedules of people and quality of life during the pandemic. The dataset is available as Supplementary Material.

### Measures

#### Protection motivation

The PMT constructs were measured via an ad hoc Protection Motivation Questionnaire (PMQ) prepared for this study. First, a literature review of previous PMT studies was conducted to analyze the relevant measures. On this basis, the items were developed by the authors. After that, two experts (psychologists familiar with the PMT and the aim of the current study) critically reviewed the items and confirmed their content validity. The final version of the PMQ contains ten items. The participants answered using a 7-point scale (1-definitely not, 7-definitely yes). The items were averaged across the subscales. The PMQ items are presented in Table 1.

**Table 1:** The items of the Protection Motivation Questionnaire.

Construct	Statement
**Perceived vulnerability**	Compared to others, I am more vulnerable to contracting the coronavirus.
	The disease caused by the coronavirus is a bigger threat to me than to most people.
**Perceived severity**	The disease caused by the coronavirus is dangerous.
	The disease caused by the coronavirus has serious health implications.
**Perceived self-efficacy**	Remembering to observe the more stringent hygiene rules associated with the coronavirus is not a problem for me.
	I am capable of remaining in isolation for as long as necessary.
**Perceived response-efficacy**	I believe that social distancing can protect against infection.
	I believe that following the hygiene rules proposed by medical authorities effectively protects people against infection.
**Perceived response costs**	Isolation from others makes everyday life very difficult and unbearable.
	The hygiene rules suggested for the time of the pandemic are extremely onerous.

*Threat appraisal*. The threat appraisal scale, which evaluates the perception of the potential risk of threat, consists of the items which measure subjective vulnerability to COVID-19 infection (2 items, α = 0.82) and subjective severity of the illness (2 items, α = 0.87).

*Coping appraisal*. The coping appraisal scale was intended to measure the perception of protective responses against threats associated with the pandemic. This scale contains the items measuring self-efficacy, i.e., the opinion whether the individual will manage to employ protective behaviors (2 items, α = 0.45), response-efficacy, i.e., the opinion whether protective measures can be effective against the threat (2 items, α = 0.71) and response costs, i.e., costs for the individual which can be an effect of taking protective behavior (2 items, α = 0.46).

*Factorial structure*. According to Norman et al. ([24] p.101), confirmatory factor analysis should be performed on the PMT measures. Therefore, we performed CFA, which showed that our data fitted well to the assumed five-factor structure of the questionnaire (*χ^2^* = 34.3, *p* = 0.10, RMSEA = 0.03, CFI = 0.99, NFI = 0.97 GFI = 0.99).

*Fear of COVID-19*. An affective reaction associated with threat appraisal was measured with the Polish version of the Fear of COVID-19 scale (FCV-19S) [22, 55]. The tool contains 7 items (α = 0.89; "I am afraid of losing my life because of coronavirus 19") with answers on a 5-point scale (1-strongly disagree, 5-strongly agree). The FCV-19S was translated into many languages. The Polish version demonstrated a clear factorial structure and good psychometric properties [22]. The items were averaged.

#### Coping behavior

The Coping Behavior Scale (CBS) was constructed for the current study. Participants were asked to assess whether they had behaved in particular ways during the preceding two weeks, using a 7-point scale (1- definitely not, 7-definitely yes). Following the PMT tradition, adaptive and maladaptive forms of preventive behavior were taken into account. The CBS items are presented in S1 Table.

**Adaptive coping behavior** (i.e., preventive behavior) was measured with a set of items describing adaptive behaviors widespread in Poland during the phase of the epidemic when this study was conducted. First, a pool of 25 items was generated based on information from national media (such as websites, newspapers, TV, radio) containing pieces of advice on how to avoid contracting COVID-19 during the pandemic. Then, a group of specialists (psychologists and medical doctors) reviewed the list and chose the behaviors which were widespread enough in Poland during that period to provide response variability. The participants were asked to what extent the statements described their behaviors (associated with the pandemic) during the preceding two weekends (e.g., "Keeping physical distance from other people in public places," "Disinfecting items (surfaces, door handles, phones)." The final version of the scale had ten items (α = 0.88). The items were averaged.

**Maladaptive coping behavior** was measured with six items and regarded avoidant behavior (3 items, α = 0.76; " Avoiding thinking about the threat posed by the pandemic") and wishful thinking (3 items, α = 0.69; " Explaining to yourself that the coronavirus causes a disease not much more dangerous than the flu".) The items were developed by the authors on the basis of the PMT literature. The items were averaged across the subscales.

*Factorial structure*. CFA was performed to confirm the assumed three-factor structure of the CBS. The data fitted the model well (*χ^2^* = 24.17, *p* = 0.044, RMSEA = 0.04, CFI = 0.99, NFI = 0.98 GFI = 0.99).

#### Personality traits

Personality traits were measured with the International Personality Item Pool–Big Five Markers–20 questionnaire (IPIP-BFM-20 [56]), the short Polish version of the IPIP-BFM-50 (www.ipip.ori.org, [57]). The questionnaire contains 20 items assessing the Big Five personality traits, with the answers on a five-point scale (1-very inaccurate, 5-very accurate). The IPIP-BFM-20 was used in a number of published studies presenting good psychometric properties. In the current study, the reliability of the subscales was acceptable (extraversion, α = 0.87, agreeableness, α = 0.70, conscientiousness, α = 0.76, neuroticism, α = 0.75, intellect, α = 0.66).

### Statistical analyses

IBM SPSS (version 26) and JASP (version 13.1) were used to analyze the data. First, descriptive statistics and correlations between variables were computed. Student’s t-test was used to assess gender differences. To identify factors associated with coping behavior, correlational analysis was performed to determine relationships between each of the PMT constructs and the B5 personality traits on the one hand and coping behavior during the pandemic on the other hand. Cohen’s [58] classification was used to interpret effect size: coefficients of 0.1 represent weak associations, those of 0.3 represent medium associations, and those of 0.5 describe strong associations. After that, two separate multiple regression analyses were performed to assess the total predictive value of the PMT constructs as well as the B5 traits in predicting coping when the other PMT or B5 variables were statistically controlled. VIFs were < 1.5, and tolerance was < 1, indicating that multicollinearity of the predictors is not a large problem in these analyses. Pairwise deletion was used to handle missing data.

Then, a hierarchical regression analysis was performed to assess the incremental validity of the PMT constructs above the B5 personality traits and sociodemographic factors. Because significant deviation from normality was detected for age (skewness = 1.4), this variable was log-transformed prior to the analyses. All the predictors were standardized. We followed the principles regarding the proper hierarchical order of predictor entry, formulated by Cohen and Cohen [59]. The static variables (age and gender) were entered simultaneously in Step 1, followed by personality variables entered as a block in Step 2, and the PMT constructs entered as a block in Step 3. This was in line with the presumed causal priority principle because personality traits (which are heritable and relatively stable across the lifespan) can be the potential causes of any cognitive-emotional evaluations (such as those reflected in the PMT constructs). The block method of entering was used because the aim of this analysis was to examine the contribution of the two groups of variables rather than the particular predictors.

## Results

### Preliminary analyses

In Table 2, descriptive statistics and correlations for study variables are displayed. The participants reported higher levels of adaptive (preventive) behavior (*M* = 5.05, *SD* = 1.2) than maladaptive behavior (avoidant behavior *M* = 3.43, *SD* = 1.5, wishful thinking *M* = 3.09, *SD* = 1.3). The majority of the participants (80%) had a mean score on adaptive behavior higher than four. Two maladaptive coping strategies correlated moderately, whereas preventive behavior was associated negatively, weakly, with wishful thinking. Age correlated with the predictors of coping behavior (positively with perceived vulnerability, perceived costs, fear of COVID-19, conscientiousness, and extraversion, and negatively with self-efficacy and neuroticism), but these relationships were weak (range -.17 –.28). The correlations of age with coping behaviors were non-significant. There were significant differences between women and men in maladaptive coping behavior (avoidant behavior *t* = 6.80 *p*<0.001; wishful thinking *t* = 3.86, *p*<0.001), fear of COVID-19 (*t* = 5.52, *p*<0.001), agreeableness (*t* = 3.44, *p* = 0.001), and conscientiousness (*t* = 4.35, *p*<0.001). In either case, women had higher scores than men.

**Table 2:** Descriptive statistics and intercorrelations between the variables.

Variable	(1)	(2)	(3)	(4)	(5)	(6)	(7)	(8)	(9)	(10)	(11)	(12)	(13)	(14)	(15)
**Age (1)**	-	0.28***	-0.02	0.21***	-0.17***	0	0.13**	0.09	0.20***	0.23***	-0.18***	-0.05	-0.05	-0.04	0.08
**Perceived vulnerability (2)**		-	0.26***	0.35***	0.18***	0.17***	-0.04	0.11*	0.02	0.03	0.09	-0.05	0.23***	-0.03	-0.16**
**Perceived severity (3)**			-	0.37***	0.33***	0.44***	-0.23***	0.13*	0.03	-0.10*	0.15**	-0.05	0.54***	-0.09	-0.39***
**Fear of COVID-19 (4)**				-	0.05	0.22***	0.09	0.18***	0.09	0.06	0.22***	-0.22***	0.42***	0.23***	0.04
**Perceived self-efficacy (5)**					-	0.40***	-0.46***	-0.01	-0.03	-0.17***	-0.04	0.11*	0.40***	-0.05	-0.26***
**Perceived response-efficacy (6)**						-	-0.36***	0.19***	0.01	-0.12*	0.08	0.05	0.55***	-0.11*	-0.21***
**Perceived costs (7)**							-	0.14**	0.08	0.36***	0.02	-0.09	-0.21***	0.21***	0.40***
**Agreeableness (8)**								-	0.24***	0.29***	0.02	0.11*	0.24***	0.07	0.00
**Conscientiousness (9)**									-	0.23***	-0.19***	0.10*	0.10*	0.10	0.11*
**Extraversion (10)**										-	-0.3***	0.17***	-0.05	0.10*	0.21***
**Neuroticism (11)**											-	0.12*	0.14**	-0.04	0.05
**Intellect (12)**												-	0.11*	-0.06	-0.05
**Preventive behavior (13)**													-	0.10	-0.20***
**Avoidant behavior (14)**														-	0.44***
**Wishful thinking (15)**															-
***M***	31.36	2.82	5.12	1.99	5.09	5.12	3.6	3.55	3.23	2.76	2.82	3.81	5.05	3.43	3.09
***SD***	11.58	1.55	1.28	0.73	1.29	1.19	1.36	0.72	0.89	1.05	0.86	0.66	1.2	1.46	1.3

*p < 0.05

**p < 0.01

***p < 0.001 (two-tailed). *N* = 397.

### Correlation analysis

All the PMT and B5 constructs correlated significantly with preventive behavior, except for extraversion (see Table 2). Among the PMT constructs, perceived costs were negatively associated with preventive behavior, whereas the remaining PMT constructs (i.e., perceived vulnerability, severity, self-efficacy, and response-efficacy) were positively related to this outcome. Among personality traits, neuroticism, agreeableness, conscientiousness, and intellect were positively associated with preventive behavior. These results provided support for H1a,b, and H3a.

Two types of maladaptive behavior showed different patterns of correlations with the PMT and B5. Wishful thinking correlated with the PMT constructs: negatively with perceived vulnerability, severity, self-efficacy, and response-efficacy and positively with perceived costs. However, wishful thinking did not correlate with fear of COVID-19, which also was predicted in this study. Thus, the results partially supported H2a,b. In turn, avoidant behavior correlated with three PMT constructs (perceived costs and fear of COVID-19 –positively and perceived response-efficacy–negatively), which is congruent with H2b and partially congruent with H2a. Among the B5 traits, extraversion and conscientiousness correlated positively with wishful thinking; only extraversion correlated (positively) with avoidant behavior. Neuroticism did not correlate with maladaptive coping behavior; thus, H3b did not receive support.

### Multiple regressions

#### The PMT models

The results of multiple regression analyses are presented in Table 3. When analyzed together, the PMT variables explained 48% of the protective behavior variance (Model 1A), 12% of avoidant behavior (Model 2A), and 29% of wishful thinking (Model 3A). For preventive behavior, the model was significant (*F*(6,380) = 59.7, *p*<0.001) and perceived severity, self-efficacy, response-efficacy, and fear of COVID-19 emerged as its significant positive predictors. For avoidant behavior, the model was significant (*F*(6,380) = 8.8, *p*<0.001). Perceived costs and fear of COVID-19 were positive predictors of avoidant behavior, whereas perceived severity was a negative predictor. For wishful thinking, the model was significant (*F*(6,380) = 25.7, *p*<0.001), and the outcome was predicted by perceived vulnerability and severity (negatively) and perceived costs and fear of COVID-19 (positively).

**Table 3:** Multiple linear regression.

	Model 1		Model 2		Model 3	
	Preventive behavior		Avoidant behavior		Wishful thinking	
	*β*	p-value	*β*	p-value	*β*	p-value
**Models A**						
**Perceived vulnerability**	-.013	.749	-.097	.064	-.130	.006
**Perceived severity**	.259	< .001	-.135	.021	-.367	< .001
**Fear of COVID-19**	.242	< .001	.310	< .001	.185	< .001
**Perceived self-efficacy**	.198	< .001	.101	.081	-.001	.989
**Perceived response-efficacy**	.320	< .001	-.081	.161	.048	.356
**Perceived costs**	.038	.377	.162	.005	.314	< .001
***R*** ^***2***^	0.48		0.12		0.29	
**Models B**						
**Agreeableness**	.242	< .001	.029	.587	-.078	.139
**Conscientiousness**	.080	.116	.088	.098	.088	.093
**Extraversion**	-.121	.024	.109	.052	.231	< .001
**Neuroticism**	.122	.018	.073	.172	.029	.584
**Intellect**	.109	.027	-.084	.099	-.089	.076
***R*** ^***2***^	0.10		0.03		0.06	

#### The B5 models

When analyzed together, the B5 traits explained 10% of the preventive behavior variance (Model 1B), 6% of wishful thinking (Model 2B), and 3% of avoidant behavior (Model 3B; see Table 3). For preventive behavior, the model was significant (*F*(5,391) = 8.8, *p*<0.001). Agreeableness, neuroticism, and intellect (positively), and extraversion (negatively) predicted preventive behavior. For avoidant behavior, the model was significant (*F*(5,391) = 2.36, *p* = 0.039); however, none of the predictors reached statistical significance. For wishful thinking, the model was significant (*F*(5,391) = 5.0, *p*<0.001), and extraversion emerged as a significant positive predictor of the outcome.

### Hierarchical regression

The predictors were entered in three blocks to determine incremental validity of the PMT constructs above and beyond sociodemographic and personality traits in predicting coping behavior. The effects of the predictors on preventive behavior (Table 4), avoidant behavior (Table 5), and wishful thinking (Table 6) were tested.

**Table 4:** Results of hierarchical regressions with the PMT constructs and personality traits predicting together adaptive behavior during the pandemic.

Predictors	Step 1				Step 2				Step 3			
	*B*	*SE*	*β*	p-value	*B*	*SE*	*β*	p-value	*B*	*SE*	*β*	p-value
**Age**	-.06	.06	-.05	.342	-.03	.06	-.02	.627	-.08	.05	-.06	.121
**Gender**	-.17	.06	-.14	.005	-.11	.06	-.09	.077	-.05	.05	-.04	.295
**Agreeableness**					.28	.06	.24	< .001	.10	.05	.09	.033
**Conscientiousness**					.08	.06	.06	.221	.06	.05	.05	.220
**Extraversion**					-.15	.06	-.13	.019	-.03	.05	-.03	.559
**Neuroticism**					.12	.06	.10	.058	.03	.05	.03	.523
**Intellect**					.14	.06	.12	.019	.16	.04	.14	< .001
**Perceived vulnerability**									.01	0.5	.01	.917
**Perceived severity**									.30	.05	.25	< .001
**Fear of COVID-19**									.30	.05	.25	< .001
**Perceived self-efficacy**									.21	.05	.18	< .001
**Perceived response-efficacy**									.35	.05	.29	< .001
**Perceived costs**									.03	.05	.02	.596
***R***^***2***^ ***/ ΔR***^***2***^	0.02 / -				0.11 / 0.09				0.52 / 0.41			

**Table 5:** Results of hierarchical regressions with the PMT constructs and personality traits predicting together avoidant behavior during the pandemic.

Predictors	Step 1				Step 2				Step 3			
	*B*	*SE*	*β*	p-value	*B*	*SE*	*β*	p-value	*B*	*SE*	*β*	p-value
**Age**	-.15	.07	-.10	.034	-.19	.07	-.13	.010	-.23	.08	-.16	.003
**Gender**	-.49	.07	-.33	< .001	-.46	.07	-.32	< .001	-.39	.07	-.27	< .001
**Agreeableness**					-.00	.07	-.00	.974	-.00	.07	-.00	.968
**Conscientiousness**					.05	.08	.04	.477	.04	.07	.03	.541
**Extraversion**					.14	.08	.10	.064	.02	.08	.01	.789
**Neuroticism**					.00	.08	.00	.971	-.07	.08	-.05	.380
**Intellect**					-.09	.07	-.06	.191	-.00	.07	-.00	.967
**Perceived vulnerability**									-.05	.08	-.04	.484
**Perceived severity**									-.21	.08	-.15	.010
**Fear of COVID-19**									.38	.08	.26	< .001
**Perceived self-efficacy**									.08	.08	.06	.307
**Perceived response-efficacy**									-.09	.08	-.06	.255
**Perceived costs**									.19	.08	.13	.027
***R***^***2***^ ***/ ΔR***^***2***^	0.11 / -				0.12 / 0.01				0.20 / 0.08			

**Table 6:** Results of hierarchical regressions with the PMT constructs and personality traits predicting together wishful thinking during the pandemic.

Predictors	Step 1				Step 2				Step 3			
	*B*	*SE*	*β*	p-value	*B*	*SE*	*β*	p-value	*B*	*SE*	*β*	p-value
**Age**	.05	.07	.04	.745	-.02	.07	-.01	.803	-.02	.06	-.01	.789
**Gender**	-.24	.07	-.18	< .001	-.22	.07	-.17	.001	-.18	.06	-.14	.002
**Agreeableness**					-.12	.07	-.10	.068	-.10	.06	-.08	.115
**Conscientiousness**					.07	.07	.05	.338	.07	.06	.06	.221
**Extraversion**					.29	.07	.22	< .001	.08	.07	.07	.209
**Neuroticism**					-.00	.07	-.00	.946	-.03	.06	-.02	.669
**Intellect**					-.10	.06	-.08	.134	-.03	.06	-.02	.636
**Perceived vulnerability**									-.15	.06	-.12	.016
**Perceived severity**									-.47	.07	-.36	< .001
**Fear of COVID-19**									.19	.07	.15	.005
**Perceived self-efficacy**									-.01	.07	-.01	.882
**Perceived response-efficacy**									.09	.07	.07	.188
**Perceived costs**									.37	.07	.29	< .001
***R***^***2***^ ***/ ΔR***^***2***^	0.04 / -				0.09 / 0.05				0.32 / 0.23			

For preventive behavior, sociodemographic variables (entered in step 1) accounted for a significant amount of variance (*R*^2^ = 0.02, *F*(2,384) = 4.0, *p* = 0.019), but only gender became a significant predictor (see Table 4). Being female was associated with higher levels of preventive behavior. The B5 traits (entered in step 2) accounted for a significant increase in the variance of preventive behavior beyond age and gender (Δ*R^2^* = 0.09, *p*<0.001; *F*(7,379) = 6.7, *p*<0.001). Agreeableness, intellect, and extraversion were found to contribute significantly to this change in the outcome variance. Lower extraversion and higher agreeableness and intellect were associated with higher levels of preventive behavior. The PMT constructs (entered in step 3) accounted for an increase in preventive behavior variance beyond sociodemographic and personality variables (Δ*R^2^ =* 0.41, *p*<0.001; *F*(13,373) = 31.3, *p*<0.001). Perceived severity, self-efficacy, response-efficacy, and fear of COVID-19 were significant predictors of the outcome. Higher scores on these predictor variables were associated with higher levels of preventive behavior. In this final model, also agreeableness and intellect remained significant.

For avoidant behavior, sociodemographic variables (entered in step 1) accounted for a significant amount of variance (*R^2^ =* 0.11, *F*(2.384) = 23.9, *p*<0.001), and age and gender were significant predictors (see Table 5). Lower age and being female were associated with higher scores on avoidant behavior. The B5 traits (entered in step 2) did not account for a significant increase in the variance of avoidant behavior beyond sociodemographic variables (Δ*R^2^ =* 0.01, n.s.; *F*(7,379) = 7.67, *p* = <0.001) and none of the B5 traits emerged as a significant predictor of the outcome. The PMT constructs (entered in step 3) accounted for an increase in avoidant behavior variance beyond sociodemographic and personality variables (Δ*R^2^ =* 0.08, *p*<0.001; *F*(13,373) = 7.2, *p*<0.001). Perceived severity, fear of COVID-19, and perceived costs appeared as significant predictors of avoidant behavior. Higher scores on fear of COVID-19 and perceived costs and lower perceived severity of illness were associated with higher levels of avoidant behavior. In the final model, age and gender remained significant predictors of avoidant behavior.

For wishful thinking, sociodemographic variables (entered in step 1) accounted for a significant amount of its variance (*R^2^ =* 0.04, *F*(2,384) = 7.4, *p* = 0.001), but only gender was a significant predictor (see Table 6). Being female was associated with higher scores on wishful thinking. The B5 traits (entered in step 2) accounted for a significant increase in the variance of wishful thinking beyond sociodemographic variables (Δ*R^2^ =* 0.05, *p* = 0.001; *F*(7,379) = 5.2, *p*<0.001). However, only extraversion emerged as its significant predictor. Higher extraversion was associated with a higher tendency towards wishful thinking. The PMT constructs (entered in step 3) accounted for an increase in wishful thinking variance beyond sociodemographic and personality variables (Δ*R^2^ =* 0.23, *p*<0.001; *F*(13,373) = 13.4, *p*<0.001). Perceived vulnerability, severity, costs, and fear of COVID-19 were identified as significant predictors. Higher perceived costs and fear of COVID-19 as well as lower perceived vulnerability and severity were associated with higher scores on wishful thinking.

The final regression models for coping behavior during the pandemic explained 52% of the variance in preventive behavior, 20% of the variance in avoidance behavior, and 32% of the variance in wishful thinking.

## Discussion

Three sets of behaviors related to the pandemic have been investigated: preventive behavior (i.e., following the pandemic rules and restrictions), avoidant behavior (i.e., coping with stress and uncertainty by trying to avoid talking, thinking, or receiving information about the pandemic), and wishful thinking (i.e., unrealistic optimism regarding the pandemic and its effects). Avoidant behavior and wishful thinking were regarded as maladaptive in this study because they do not contribute to reducing the risk of contracting the coronavirus (although they can lower stress and anxiety). The participants reported adaptive (preventive) behaviors more frequently than avoidant behavior or wishful thinking: the mean for preventive behavior was about 5, that is above the midpoint of the 7-point scale, whereas the means for maladaptive behaviors were below the midpoint of the scale (between 3 and 3.5). The majority of participants (80%) declared that they had generally engaged in preventive behavior during the preceding two weeks, which is congruent with the results of other studies conducted in the same period in Poland [60]. The two forms of maladaptive coping correlated moderately in our study, which can suggest that a preference to use them can co-occur. On the other hand, such co-occurrence was not present in the case of adaptive and maladaptive behavior, as no correlation between preventive and avoidant behavior and negative (weak) correlation between preventive behavior and wishful thinking were observed.

The primary aim of this study was to evaluate the predictive value of the constructs proposed by two influential theoretical frameworks (the PMT and the B5 model of personality) in predicting adaptive and maladaptive coping behavior during the SARS-Cov-2 pandemic. Correlational and regression analyses enabled establishing the relationships between coping behavior and each predictor variable separately, as well as assessing the total predictive value of the B5 and PMT models and incremental validity of the PMT constructs above sociodemographic variables and the B5 personality traits.

### PMT constructs and adaptive behavior

The results of the correlational analysis showed that all the PMT constructs were significantly associated with preventive behavior, and the direction of these relationships (perceived costs–a negative relationship, the remaining PMT variables–positive relationships) was congruent with all our predictions formulated on the basis of the PMT model. The appraisal of the severity of the threat associated with the SARS-Cov-2 pandemic was based on evaluations of personal vulnerability or susceptibility to the health threat (i.e., coronavirus infection), perceived general severity of a COVID-19 disease, and fear of COVID-19. Of these three constructs, perceived severity of illness was the strongest predictor of preventive behavior (*r* = 0.54), followed by fear of COVID-19 (*r* = 0.42) and perceived vulnerability to the coronavirus (*r* = 0.23). In turn, the coping appraisal included three constructs (i.e., self-efficacy, response-efficacy, and perceived costs of preventive behavior). In the current study, self-efficacy was defined as a belief that one was able to cope with health threats posed by the coronavirus. In turn, response-efficacy assumed the belief that remedies proposed by health authorities could be effective and protect the individual from infection with the coronavirus, serious illness, or death. As in the case of the severity appraisal, the perceived response-efficacy, unrelated to individual dispositions, was more strongly associated with preventive behavior (*r* = 0.55) than the evaluations of the individual’s self-efficacy (*r* = 0.40). If a person believes that the recommended preventive measures are effective against the coronavirus, their motivation to follow them strengthens, which was reflected in this result. In turn, the perceived costs of preventive actions were only weakly associated with preventive behavior (*r* = -0.21). This could be the case because the study was conducted during the first wave of the pandemic when people hoped the pandemic would end very soon, which could influence their evaluations of the costs. Generally, these results are congruent with other findings that underline the importance of the belief that protective measures (recommended by health authorities and governments during the pandemic) can be effective [61].

Our findings are also in line with other studies that used the PMT constructs to investigate motives to protect oneself (and others) as well as epidemic-related behavior during the COVID-19 pandemic. For example, Bashirian et al. [35] found that both threat and coping appraisal were significant predictors of protection motivation in nurses in Iran. Similar results were also obtained by Jørgensen et al. [37] on community samples from eight countries, Farooq et al. [41] on a sample from Finland, or Rad et al. [62] on a community sample from Iran. However, in some studies, only coping appraisal (but not threat appraisal) predicted protective behavior during the pandemic [36].

### PMT constructs and maladaptive behavior

The results of correlational analysis on maladaptive behavior and the PMT constructs revealed different patterns of associations. Five out of six PMT variables were significantly associated with wishful thinking, and all these relationships were in the opposite direction compared to preventive behavior. A tendency towards wishful thinking was higher when threat appraisal and coping appraisal constructs (except perceived costs) were lower. According to the PMT, wishful thinking, avoidance, and other maladaptive behaviors can occur when the individual perceives a threat [28]. The current results are different because of the negative correlation between wishful thinking and the elements of threat appraisal (i.e., perceived vulnerability and severity) and the lack of correlation with the fear of COVID-19. On the other hand, the negative associations between wishful thinking and perceived response-efficacy and self-efficacy and a positive relationship between wishful thinking and perceived costs were congruent with the PMT and empirical evidence [28, 49, 50, 63]. In our sample, relatively the strongest relationship was the positive relationship between perceived costs and wishful thinking (*r* = 0.40). This suggests that perceiving personal costs of following pandemic recommendations as high (e.g., when one believes that following the pandemic rules can make their personal needs difficult to meet) can push the individual towards such coping strategies as mistaken or unrealistic beliefs and self-deception, regardless of the level of fear. Alternatively, unrealistic optimism at this stage of the pandemic could perhaps be an effective strategy as a way to reduce fear in a novel and deeply uncertain situation. However, it is worth noting that after controlling for the other PMT constructs, fear of COVID-19 emerged as a significant albeit weak (*β* = 0.18) predictor of wishful thinking.

Avoidant behavior–the second type of maladaptive behavior measured in this study, was positively associated with perceived costs and fear of COVID-19 and negatively associated with perceived response-efficacy; however, these associations were weak. It seems that the emergence of avoidant behavior in the context of the pandemic could be more frequent in persons with higher levels of the fear of COVID-19 who tended to perceive the pandemic measures as less effective and the costs of following them as relatively high. This result is in line with the PMT and previous studies [24, 52].

### The predictive value of the PMT constructs in predicting coping behavior

Multiple regression analysis enabled establishing the relative importance of the PMT constructs when their common variance was statistically controlled (see Table 3). Perceived response-efficacy emerged as the strongest (positive) predictor of preventive behavior, and the PMT constructs explained together 48% of its variance. The fear of COVID-19 was the strongest (positive) predictor of avoidant behavior, and the PMT constructs predicted 12% of the variance in this type of behavior. In turn, perceived severity turned out to be the strongest (negative) predictor of wishful thinking and predicted, together with the other PMT variables, 29% of its variance.

In the current study, some differences in the predictive value of the particular PMT constructs were obtained. In the meta-analyses [28, 29], coping appraisal variables showed higher predictive validity than threat appraisal constructs in predicting health-related behavior. However, in the current study, this difference was not observed in relation to adaptive behavior. In the case of maladaptive coping, threat appraisal constructs demonstrated together higher predictive validity than coping appraisal components.

During the first wave of the SARS-Cov-2 pandemic, the quick and uncontrollable spread of the virus, the lack of effective treatment, and relatively high mortality rates induced strong emotional responses in many people. In this study, these negative emotions were captured by the measure of the fear of COVID-19. The scores on this scale were positively associated with adaptive and maladaptive coping behavior. Fear can promote taking preventive actions against infections if an individual knows what they should do to avoid harm. The role of the fear of COVID-19 as an important factor facilitating the agreement with preventive measures during the pandemic was widely discussed in the literature [20, 21, 55]. However, our results showed that higher fear of COVID-19 is also related to more frequent use of maladaptive coping behaviors, which is congruent with research on the role of fear and fear appeals on behavior change [64].

### The B5 constructs and coping behavior

The results of the correlational analysis showed that all but one the B5 trait were significantly and positively associated with preventive behavior. This result was consistent with the expectations that agreeableness and conscientiousness would be predictors of adaptive coping (H3a) and with other studies on protective behavior [5, 6, 15, 16, 18]. In multiple regression, the B5 traits predicted together 10% of the variance in preventive behavior. However, controlling for the shared variance caused that conscientiousness lost significance and extraversion emerged as a negative predictor of preventive behavior. Wishful thinking and avoidant behavior correlated weakly with extraversion. Additionally, very small correlations with conscientiousness were observed. Thus, H3b, which stated that neuroticism would be a positive predictor of maladaptive coping behavior, was not supported by the data.

In general, the B5 traits had minimal predictive value for maladaptive coping behavior. All the B5 traits predicted 6% of the variance in wishful thinking and 3% of the variance in avoidant behavior.

In multiple regression analysis, extraversion was the only significant predictor of wishful thinking and (at a statistical trend level) avoidant behavior and a negative predictor of preventive behavior. These associations can be explained in reference to the core features attributed to extraversion, such as a higher need for stimulation, sociability, behavioral activity, and risk-taking [65, 66]. During the Sars-Cov-2 pandemic, social distancing and isolation–the key anti-pandemic measures–caused severe limitations in social contacts, which may be especially difficult to accept for extraverts. In a study by Liu et al. [67] extraversion was related to higher distress during the pandemic, and this relationship was not mediated by perceived threat and efficacy. For extraverts, the possibility to fulfill their social needs may be hindered during a pandemic. Thus, adaptive responses can be viewed as especially costly, and prohibited activities, such as socializing, can be perceived as especially rewarding. Instead of following pandemic rules and isolate themselves from others, extraverts could try to reduce fear using such strategies as wishful thinking or avoidant behavior. What is more, extraversion is strongly associated with optimism, which–especially in people with low self-efficacy–can lead to so-called "defensive optimism" associated with threat avoidance [68] and maladaptive coping. The negative relationships between extraversion and adherence to pandemic-related measures were previously found in some of the studies on pandemic behavior [69–71].

In the final analysis, hierarchical regression was performed. The PMT variables showed incremental validity over and above sociodemographic variables and personality traits in predicting both adaptive and maladaptive coping behavior. The final models accounted for 52% of the variance in preventive behavior, 20% of the variance in avoidance behavior, and 32% of the variance in wishful thinking.

## Limitations and conclusions

This study has several limitations. The data were collected via self-report, which is not optimal, especially when adherence behavior is measured. Our study had a cross-sectional design; thus, only concurrent preventive behavior can be analyzed, and causal inferences cannot be drawn. The study was conducted as an online survey; so persons without access to the Internet were unable to participate. The alpha reliability values of the two subscales of the PMQ (i.e., response costs and self-efficacy) were low. This may be the case when a scale contains few items, because the Cronbach’s alpha depends, among others, on the number of items [72]. Additionally, our items were not formulated with the aim of optimizing internal consistency, but rather content validity of the scale was a priority [73]. The current study was conducted in difficult pandemic times. To minimize burden on participants, our measure of the PMT constructs was short. Nevertheless, future studies should consider using longer measures of the PMT variables.

In conclusion, this study added to the PMT literature on health-related coping behavior by giving empirical evidence from an East-Central European population. The findings provided strong support for the PMT predictive value, especially for adaptive (preventive) behavior during the Sars-CoV-2 pandemic. The B5 personality traits accounted for a small proportion of the variability of pandemic-related coping behavior, especially when maladaptive behavior was the outcome. Our findings showed that the PMT model had incremental validity over demographic variables and personality traits.

This study also had some practical implications. According to the current results, to increase adherence to preventive measures during an epidemic, all the PMT constructs should be considered while persuasive communication to the public is formulated. Both threat appraisal and coping appraisal cognitions could be shaped by such communication. The fear appeals alone may not be effective because higher levels of fear can lead to both adaptive and maladaptive coping, which was visible in our findings. In turn, maladaptive coping may hinder protection motivation and actions [51]. Presenting effective ways to avoid infection and enhancing the sense of personal efficacy can be a way to manage this problem.

## Supporting information

S1 Table The items of the Coping Behavior Scale. (PDF)

S1 Database Dataset. (SAV)
