# Supplementary material for: The predictors of adaptive and maladaptive coping behavior during the COVID-19 pandemic: The Protection Motivation Theory and the Big Five personality traits
Source: PLoS One. 2021 Oct 19;16(10):e0258606. doi: 10.1371/journal.pone.0258606 (PMC8525766; doi:10.1371/journal.pone.0258606)
Supplement: S1 Table — (PDF) [file pone.0258606.s001.pdf]

S1 Table. **The items of the Coping Behavior Scale.** *(Did you engage in any of the following activities during the previous two weeks?)*

Construct	Statement
**Preventive behavior**	Staying at home, limiting going out only to cases in which it is necessary.
	Keeping physical distance from other people in public places.
	Switching from public transport to driving, cycling, etc.
	Not socializing with friends/family.
	Shopping during less busy hours to avoid crowded places.
	Avoiding physical contact with older people.
	Disinfecting items (surfaces, door handles, phones).
	Wearing face masks.
	Frequently washing and/or disinfecting hands.
	Paying by card to minimize contact with germs and viruses.
**Avoidant behavior**	Avoiding thinking about the threat posed by the pandemic.
	Avoiding conversations about the pandemic.
	Avoiding programs and media coverage about the pandemic.
**Wishful thinking**	Explaining to yourself that the risk of contracting the coronavirus is very low in Poland.
	Explaining to yourself that perhaps the pandemic will die out on its own in the summer.
	Explaining to yourself that the coronavirus causes a disease not much more dangerous than the flu.
